# PDGFR Signaling Mediates Hyperproliferation and Fibrotic Responses of Subsynovial Connective Tissue Cells in Idiopathic Carpal Tunnel Syndrome

**DOI:** 10.1038/s41598-017-16443-w

**Published:** 2017-11-23

**Authors:** Yuki Saito, Takako Chikenji, Yasuhiro Ozasa, Mineko Fujimiya, Toshihiko Yamashita, Anne Gingery, Kousuke Iba

**Affiliations:** 1 Department of Anatomy, Sapporo Medical University School of Medicine, Sapporo, Japan; 2 Department of Orthopaedic Surgery, Sapporo Medical University School of Medicine, Sapporo, Japan; 3 Department of Orthopedic Surgery, Mayo Clinic, Rochester, United States

## Abstract

Fibrosis of the subsynovial connective tissue (SSCT) is a pathognomonic change in carpal tunnel syndrome (CTS). Identification of molecular targets and anti-fibrotic therapies could provide new treatment strategies for CTS. The contribution of SSCT cells to fibrosis and the signaling pathways that initiate and aggravate fibrosis in CTS remain unknown. Here we report that platelet-derived growth factor receptor alpha (PDGFRα) positive ( + ) cells accumulate in CTS SSCT and that the presence of fibrotic growth factor, PDGF-AA, results in increased proliferation of PDGFRα+ cells via PI3K/Akt signaling pathway. Although PI3K inhibition decreased proliferation, there was no change in fibrosis-related gene expression. Indeed, protein levels of fibrosis signaling mediator TGF-β remained the same and the second messenger, Smad2/3, accumulated in the nucleus. In contrast AMP-activated protein kinase (AMPK) activation, which can be induced with metformin and AICAR inhibited proliferation, TGF-β expression, and altered cell morphology in SSCT cells. Further we show that AMPK activation by metformin reduced collagen III levels and the ratio of Collagen I to Collagen III. Both AICAR and metformin reduced F-actin and significantly reduced the fiber cross alignment. Our results suggest that PDGFRa signaling may be an important fibrosis target and that activators of AMPK, may be an important therapeutic approach for treating CTS.

## Introduction

Carpal tunnel syndrome (CTS) is the most common compression neuropathy with incidence reported to be 2–5% in the general population^1^. Middle-age represents the peak incidence of CTS, with a lifetime risk of 25% to 30%, resulting in significant quality of life and work-time impacts on the working-age population^2^. The health care costs are substantial. The total cost of CTS in the US has been reported to be $30,000 per case, or more than $10 billion per year in the US alone^3^. Little progress in CTS treatment has been made despite the cost and large impact. Surgery remains the most effective treatment, with corticosteroids injections being the main non-surgical treatment which offers temporary relief in some patients, however the mechanism of action remains unknown^4^. We and others have shown that fibrosis of subsynovial connective tissue (SSCT) in CTS patients, may be the primary pathology associated compression neuropathy of median nerve^5,6^.

Fibrosis is defined by the deposition of excess extracellular matrix (ECM), and hyperplasia of resident mesenchymal cells resulting in increased ECM deposition^7,8^. Recent studies in cardiac, liver and muscle fibrotic diseases have demonstrated that platelet-derived growth factor receptor alpha (PDGFRα) positive ( + ) mesenchymal cells increased cellular proliferation and exhibited increased pathological ECM deposition^9–11^. Furthermore blocking PDGFR signaling has been shown to have clinical efficacy against various fibrotic disease^12–14^. PDGFRα signals through several signaling pathway including PI3K-Akt and Ras-MAPK pathway and these pathways are involved in cell growth, migration, and apoptosis resistance^14,15^. Given that PDGFRα signaling is increased in fibrotic diseases, that PDGF-A is the primary isoform found in CTS fibrosis^16^, and that we have found that PDGFRα+ cells are increased in CTS^16,17^, we explored the role of PDGFRα signaling as a potential therapeutic target for CTS.

Here we report that PDGFRα+ cells accumulate in the SSCT of CTS patients and PDGFRα signaling promotes cell proliferation and the deposition of pathological type III collagen production by PDGFRα+ cells, thus acting as a crucial driver of SSCT fibrosis. We further describe, the effect of imatinib, PI3K inhibitor, MEK1/2 inhibitor and an AMPK agonist, as potential therapeutic drugs that downregulate PDGFRα signaling through PI3K/Akt/mTOR, resulting in the suppression of fibrotic genes and proliferation of SSCT cells.

## Results

### Identification of PDGFRα+ cells in SSCT

**Figure 1:**
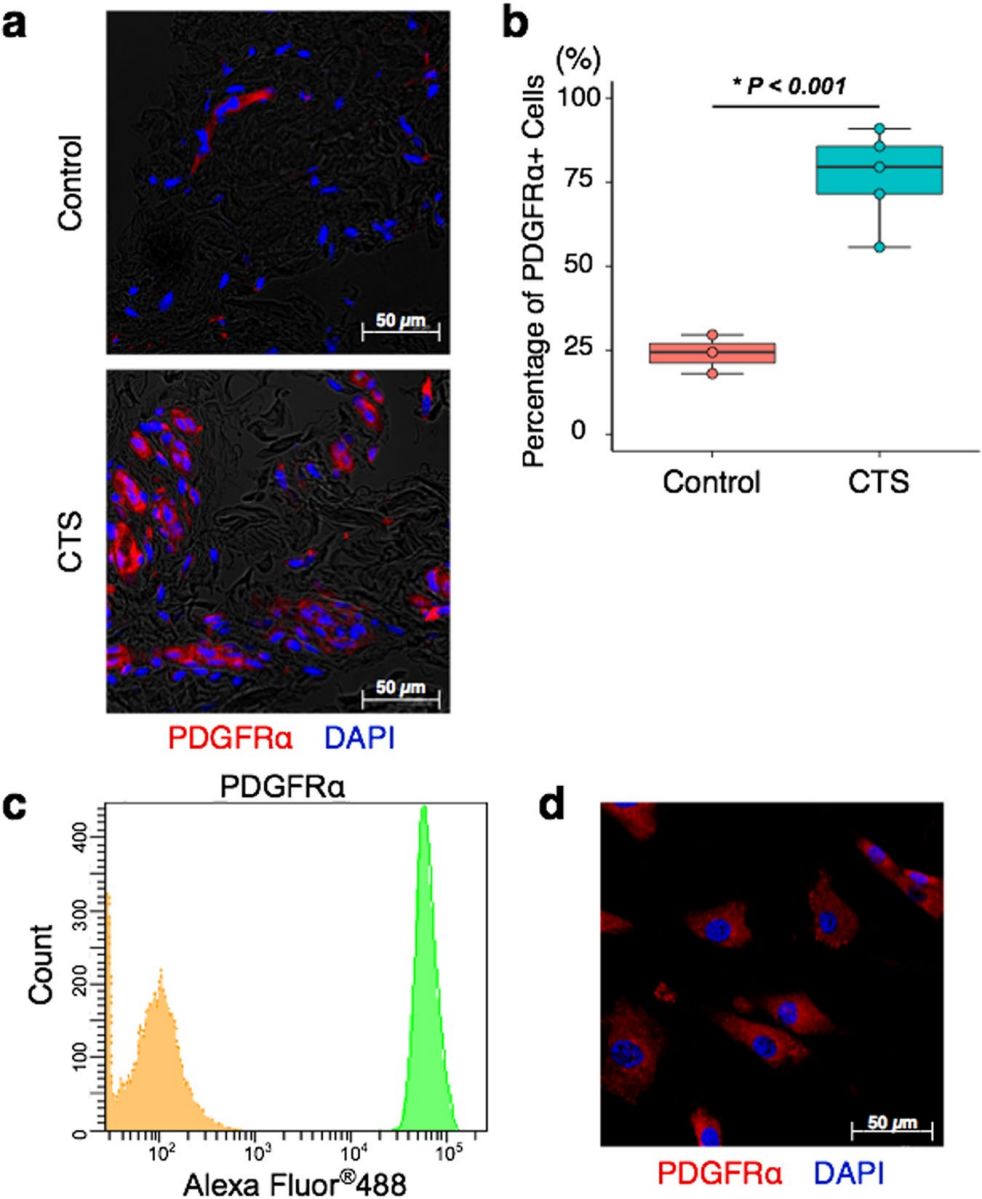
Identification of PDGFRα+ cells in SSCT. Representative image of PDGFRα+ cells identified in SSCT from CTS patients or normal controls (**a**). The percentage of PDGFRα+ cells in CTS SSCT (n = 5) were higher than that of control SSCT (n = 3) (**b**). Isolated SSCT cells from CTS expressed PDGFRα by flow cytometry (**c**) and immunocytochemistry (**d**). Boxes represent the interquartile range (IQR), lines within boxes represent the median, and lines outside boxes represent 1.5 times the IQR. Pairwise t test was used to assess the difference between control tissue and CTS tissue.

Human SSCT from CTS patients and normal subjects were evaluated for the presence of PDGFRα. We identified PDGFRα+ cells in SSCT from both CTS patients and a normal subject (Fig. 1a). Quantification of PDGFRα+ cells were determined by examining five different randomly chosen fields of view for each section. Cells were labeled and quantified by PDGFRα+ and 4′,6-diamidino-2-phenylindole (DAPI) stain. The percentage of PDGFRα+ cells (number of PDGFRα+ cells / number of DAPI) were significantly increased in CTS patients, as compared to normal subjects (*P* < 0.001; Fig. 1b).

### Isolation and characterization of SSCT cells from CTS patients

For *in vitro* experiments, the SSCT from CTS patients were digested in collagenase, and isolated cells were cultured on plastic culture dishes. Adherent cells were defined as SSCT cells. The SSCT cells were analyzed for PDGFRα expression by flow cytometry (Fig. 1c). The adherent cells expressed PDGFRα, and this was also confirmed by immunocytochemistry of cultured cells (Fig. 1d).

### Cell Proliferation and Fibrotic Gene Expression with PDGF-AA stimulation and PDGFR inhibition

**Figure 2:**
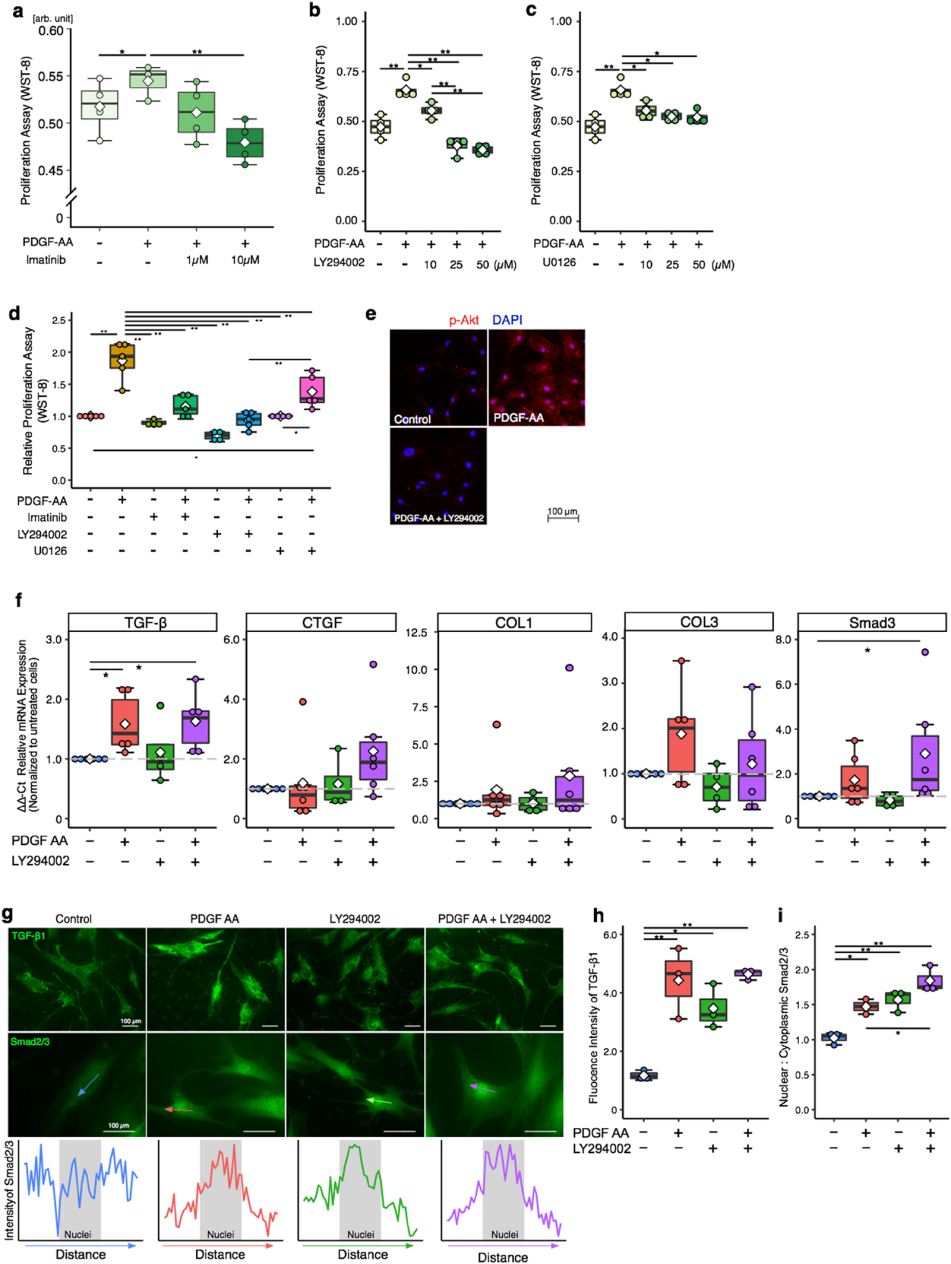
SSCT cell proliferation regulation and fibrotic gene expression after PDGF-AA stimulation and inhibition. PDGF-AA activated SSCT cell proliferation (**a**), which was inhibited by imatinib (n = 4) (**a**). The downstream signal-transduction mediators of PDGFRα action, PI3K (LY294002) and MEK1/2 (U0126) inhibitors, significantly decreased cell proliferation (**b**,**c**; n = 4). LY294002 was effective at reducing proliferation as compared to U0126 (n = 5) (**d**). Further, LY294002 inhibited Akt phosphorylation induced by PDGF-AA (**e**). Fibrotic gene expression analysis of PDGF-AA stimulated cells revealed significant increases in TGF-β, however no significant change in fibrotic gene expression with LY294002 treatment. LY294002 did not regulate Smad3 gene expressions with PDGF-AA stimulation (n = 6) (**f**). Expressions of TGFβ and localization of Smad2/3 of SSCT cells were evaluated in immunocytochemistry (**g**). Arrow indicates representative indication of where the intensity of Smad2/3 expression on nucleus was calculated. The expression of TGFβ1 (**h**; n = 4) was not changed, however nuclear localization of Smad2/3 (**i**; n = 4) was significantly enhanced by LY294002 treatment. Boxes represent the interquartile range (IQR), lines within boxes represent the median, and white rhombus represent the mean, and lines outside boxes represent 1.5 times the IQR. One-Way ANOVA was conducted to assess the difference of cell proliferation and Kruskal-Wallis test was conducted to assess the difference of relative gene expression. Pairwise comparisons were made only when the One-way ANOVA or Kruskal-Wallis test indicated statistical significance. *P*-values for multiple comparisons were adjusted by Holm methods. *P < 0.05; **P < 0.01.

In order to elucidate the effect of PDGFR signaling on SSCT cells, proliferation was evaluated by WST-8 proliferation assay. SSCT cells were treated with 10ng/mL PDGF-AA^11^. PDGF-AA stimulation significantly enhanced cell proliferation (*P* = 0.028; Fig. 2a). Imatinib, an inhibitor of PDGFRα, PDGFRβ, bcr-abl fusion protein, and c-kit^14^, inhibited proliferation of SSCT cells in a dose dependent manner (Fig. 2a). To further investigate the role of the PDGF downstream signal transduction, inhibitors LY294002 (PI3K inhibitor) and U0126 (MEK1/2 inhibitor), where used to assess the mechanism of PDGFRα action^14^. LY294002 inhibited cell proliferation at 10, 25 and 50 µM (Fig. 2b), and U0126 inhibited proliferation at 10, 25, and 50 µM (Fig. 2c). Uezumi and colleagues reported that 25 µM of LY294002 and 10 µM of U0126 inhibited PI3K and MEK1/2 which were stimulated by PDGF-AA, and that dose of inhibitor did not impair viability of human fibroblast like cells^11^. Therefore, all following inhibitor experiments use 25 µM of LY294002, 10 µM of U0126 and 10 µM of Imatinib. LY294002 significantly reduced proliferation as compared to U0126 (*P* = 0.00003; Fig. 2d) in PDGF-AA stimulated SSCT cells. Unstimulated cells treated with Imatinib, LY294002, and U0126 were not significantly changed as compared to unstimulated cells (Fig. 2d). Furthermore, we found that PI3K inhibition strongly inhibited Akt phosphorylation induced by PDGF-AA (Fig. 2e), as would be expected since Akt is downstream of PI3K. Therefore, fibrotic gene expression with PDGF-AA stimulation and inhibition of the PI3K pathway was assessed. Upon stimulation with PDGF-AA, TGF-β expression was significantly increased (*P* = 0.0083; Fig. 2f). However, inhibition of PI3K signaling with LY294002 did not regulate TGF-β expression (*P* = 0.9370; Fig. 2f). The expression of CTGF, Collagen I (Col I), Collagen III (Col III), and Smad3 were not significantly changed by PDGF-AA stimulation. Similar to TGF-β, the expression of Smad3 was statistically increased in PDGF-AA with LY294002. (*P* = 0.0083; Fig. 2f). To further confirm this finding the immunocytochemical protein expression of TGF-β was quantified and in concordance with the gene expression data LY294002 treatment did not result in changes to either TGF-β after LY294002 treatment (Fig. 2g–i). Additionally, immunocytochemical analysis of nuclear to cytoplasmic localization of Smad2/3 was evaluated here we find that LY294002 treatment of PDGF-AA treated cells resulted in increased Smad2/3 translocation (*P* = 0.0375).

### The Role of AMPK activation on cell proliferation and fibrotic gene expression

**Figure 3:**
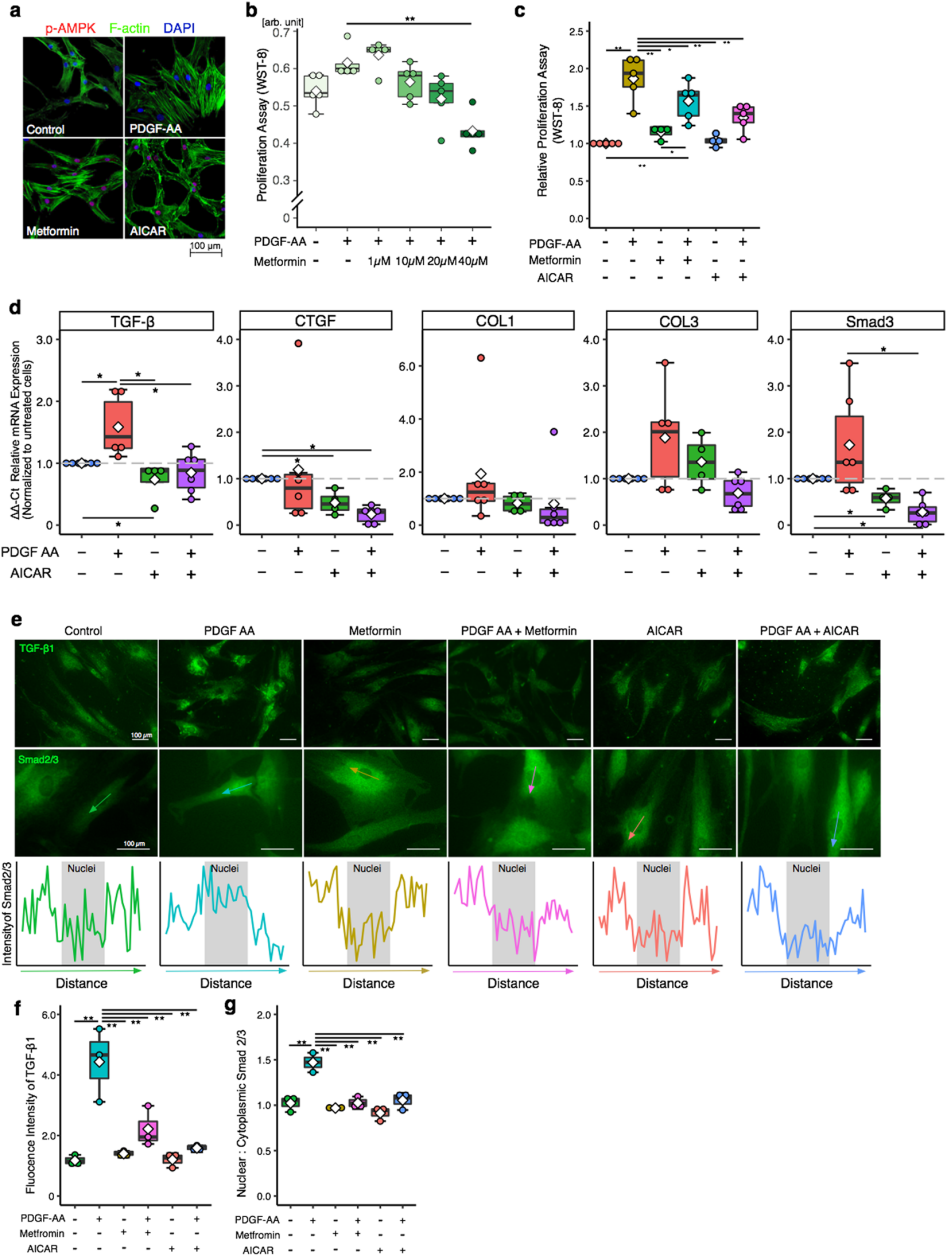
Metformin or AICAR treatment activates AMPK, decreases proliferation, and fibrotic gene expression. Metformin and AICAR activated AMPK (**a**) and inhibited cell proliferation (n = 5) (**b**,**c**). Fibrotic genes; TGF-β and Smad3 expressions were suppressed by AICAR treatment (n = 6) (**d**). Expressions of TGFβ and localization of Smad2/3 of SSCT cells were calculated in immunocytochemistry (**g**). Arrows indicate distance where was calculated the intensity of Smad2/3 expression on nucleus. TGF-β and nuclear localization Smad2/3 were significantly reduced by both metformin and AICAR treatment (**f**,**g**). Boxes represent the interquartile range (IQR), lines within boxes represent the median, and white rhombus represent the mean, and lines outside boxes represent 1.5 times the IQR. One-Way ANOVA was conducted to assess the difference of cell proliferation and Kruskal-Wallis test was conducted to assess the difference of relative gene expression. Pairwise comparisons were made only when the One-way ANOVA or Kruskal-Wallis test indicated statistical significance. *P*-values for multiple comparisons were adjusted by Holm methods. *P < 0.05; **P < 0.01.

We next examined the role of AMPK on cell proliferation since AMPK activation is known inhibit fibrotic proliferation and gene expression. AMPK coordinates cell proliferation via mTOR signaling^18^. AMPK activation was demonstrated by using 5 - aminoimidazole- 4 – carboxamide – 1 – β – D - ribofuranoside (AICAR) which is cell permeable APMK activator, and metformin which is known to activate AMPK and is widely used to treat type 2 diabetic patients^19–21^. AMPK activation was confirmed by immunocytochemistry, in both AICAR and metformin treated cells (Fig. 3a). To investigate the effect of AMPK activation on SSCT cell proliferation PDGF-AA treated cells were treated with metformin and AICAR. Metformin inhibited proliferation of SSCT cells in concentration dependent manner (*P* = 0.00003; Fig. 3b). AICAR, as well as, metformin significantly inhibited cell proliferation as compared to PDGF-AA treated cells (*P* = 0.0031 and *P* = 0.0372, respectively; Fig. 3c). Unstimulated cells treated with metformin and AICAR were not significantly changed (Fig. 3c). The effect of AMPK activation on fibrotic gene expression of PDGF-AA treated SSCT cells was evaluated in cells treated with AICAR. TGF-β and Smad3 expression was significantly decreased in AICAR treated cells as compared to PDGF-AA treated cells (*P* = 0.0173 and *P* = 0.0065, respectively; Fig. 3d). CTGF expression of AICAR treated cells was not changed upon PDGF-AA treatment, however AICAR significantly decreased CTGF gene expression as compared to non-treated control cells (*P* = 0.0083 and *P* = 0.0065, respectively; Fig. 3d). Additionally, immunocytochemistry revealed that TGF-β protein expression was inhibited by metformin and AICAR treatments with PDGF-AA stimulation (Fig. 3e,f,g). Smad2/3 nuclear translocation was significantly reduced in metformin and AICAR treatments of PDGF-AA stimulated cells (Fig. 3e,f,g).

**Figure 4:**
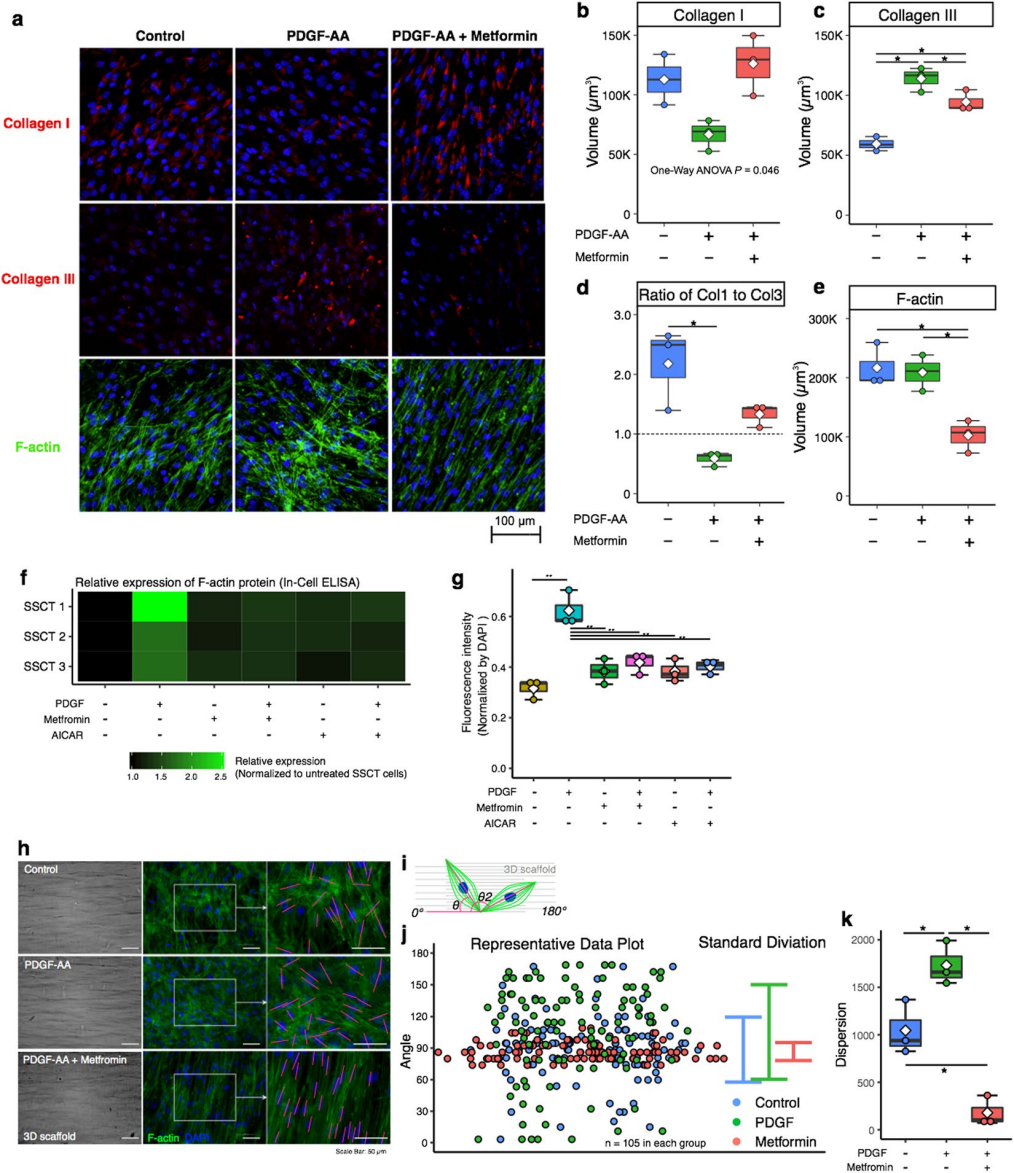
Effect on metformin on ECM production. Col I, Col III, and F-actin expression in PDGF-AA and metformin treated cells was evaluated by immunocytochemistry (**a**). PDGF-AA treated cells reduced Col I production, and increased Col III (n = 3) (**b**,**c**). The ratio of Col I to Col III in PDGF-AA treated cells was significantly reduced as compared to non-treated control cells (**d**). Metformin significantly reduced F-actin (**e**), which were oriented parallel at spindle cell shape (n = 3) (**a**). F-actin expression was quantified by In-Cell ELISA was significantly reduced by metformin and AICAR treatment (**f**,**g**). The dispersion of the F-actin in was determined (**h**). A line of F-actin which passed long axis of nucleus was defined and the calculated angle against the 3D scaffold alignment was determined (**i**) and the representative data (**j**) in each treatment. The dispersion of F-actin was decreased in metformin treated cells (**k**). Boxes represent the interquartile range (IQR), lines within boxes represent the median, and white rhombus represent the mean, and lines outside boxes represent 1.5 times the IQR. One-Way ANOVA was conducted to assess the difference among each culture conditions. Pairwise comparisons were made only when the One-way ANOVA indicated statistical significance. *P*-values for multiple comparisons were adjusted by Holm methods. *P < 0.05.

We further evaluated the effect of metformin in *ex vivo* experiments using 3D culture dishes. 3D culture dishes consist of salmon collagen-coated ePTFE (expanded polytetrafluoro- ethylene) mesh (Vecell 3D-insert). The 3D culture system was used to more accurately reflect the *in vivo* condition; allowing for long-term cell culture and evaluation of ECM deposition by immunostaining whole membrane with cells^22,23^, providing a model that allows understanding of ECM deposition as compared to 2D cultures. The volume of collagen was calculated from 3D reconstruction of laser scanning image using confocal laser scanning microscopy and NIS Elements (Nikon), and representative images were shown in supplemental Fig. 1. Using this 3D model system, we found that PDGF-AA treated cells exhibited decrease Col I and increased Col III production as compared to non-treated cells (*P* = 0.077 and *P* = 0.0006, respectively; Fig. 4a–c). PDGF-AA treated cells show decreased Col I, however treatment with metformin recovered Col I production (*P* = 0.077; Fig. 4b). Col III a marker of pathological fibrosis, was increased with PDGF-AA treatment but was significantly reduced with metformin treatment (*P* = 0.0309; Fig. 4c). The ratio of Col I to Col III, another measure of the degree of pathological fibrosis, in PDGF-AA treated cells was significantly reduced as compared to non-treated control cells, however metformin treated Col I to Col III ratios did not reach significant difference as compared to non-treated control cells (*P* = 0.0100 and *P* = 0.093, respectively; Fig. 4d). F-actin is another marker of fibrotic pathology that is increased during fibrosis. The expression is linked to cell proliferation, cell survival and ECM production via YAP/TAZ pathway, and Rho/ROCK pathway, and MRTF/SRF pathways^24–26^. F-actin upon treatment with metformin was significantly reduced as compared to both non-treated controls and PDGF-AA treated cells (*P* = 0.0140 and *P* = 0.0140, respectively; Fig. 4e). Additionally F-actin was expression was quantified by In-Cell ELISA (Fig. 4f and g). Further F-actin alignment was improved with metformin treatment as compared to normal and PDGF-AA treated cells (Fig. 4a and h). The dispersion of F-actin was calculated in each treatment (Fig. 4h–j). The dispersion was significantly decreased with metformin treatment as compared to normal and PDGF-AA treated cells (*P* = 0.0075 and *P* = 0.0005, respectively; Fig. 4k).

## Discussion

Fibrosis of the SSCT in idiopathic CTS is becoming increasingly recognized a primary pathological effector of CTS^5,6,17^. Here we identified that the PDGFRα+ cells were notably increased in SSCT of CTS patients. PDGF and PDGFRα has been implicated in fibrotic disease in many tissues^14^.

Our results revealed that PDGFRα cells were increased in SSCT of CTS patients. Yamanaka and colleagues^16^ have reported that cultured SSCT cells of CTS patients have increased PDGF-A levels upon fibrotic stimulation with TGFβ, however PDGF-B is not upregulated. Further PDGF dimers bind to the PDGF receptors with distinct binding affinity. PDGF-A binds to PDGFRα homo or heterodimers with high affinity, but does not bind to PDGFRß homodimers^14^. We, therefore examined PDGFA and PDGFRα signaling in this study.

SSCT cells derived from CTS patients increase proliferation upon PDGF-AA stimulation, and this proliferation was suppressed by inhibition of PDGFR signaling using imatinib, the PI3K inhibitor (LY294002), and MEK inhibitor (U0126). PDGFRα mediates its effect through several signaling pathways including PI3K-Akt, and Ras-MAPK. In our study, the PI3K inhibitor, LY294002, strongly decreased both cell proliferation and Akt phosphorylation after PDGF-AA, however, LY294002 either had no effect on fibrotic gene expression (TGF-β, CTGF, Col I, Col III, Smad3). TGF-β signaling regulates many cellular functions, such as cell growth, apoptosis and cell fate determination. These functions are not solely regulated by the TGF-β pathway but are also regulated by crosstalk between TGF-β and other signaling pathways. TGF-β signaling leads to the phosphorylation of the Smad2 and Smad3 C-terminal SxS motif, encouraging their interaction with Smad4 and promoting the translocation of the Smad2/3–Smad4 complexes and accumulation within the nucleus where they regulate targeted gene expression in cooperation with other cofactors. The function of this pathway is also affected by other signaling pathways. Notably, PI3K/AKT pathway has been showed to modulate TGF-β signaling through a direct interaction with Smad3^27,28^, as well as inhibiting the Smad2/3 activity by modulating their degradation via phosphorylation of a particular threonine residue within the Smad2/3 linker region^29^. Indicating that while inhibiting PI3K with LY294002, may reduce cell proliferation, fibrotic transcriptional response was not impacted.

It has been reported that activation of PDGFRα signaling stimulates Smad3 in hepatic stellate cells^30,31^, and that Smad3 promotes TGF-β autoinduction^32^. Furthermore, Smad3 signaling has been found to be regulated by PDGFRα, but not PDGFRβ, through direct binding with TGF-β receptor II^30^, indicating that PDGFRα signaling can upregulate TGF-β expression though Smad3. Further, Furukawa *et al*. reported that activation of Smad3 was stimulated by p38 MAPK^33^, which are also upregulated by PDGFRα activation though PDGF-AA stimulation^34^. Activation of PDGFRα signaling by PDGF-AA can directly stimulate Smad3 or p38 MAPK pathway and may result in activation of TGF-β.

To test whether fibrotic gene expression and cell proliferation could be suppressed concurrently, we activated AMPK by treatment with metformin or AICAR. AMPK is known as a cellular energy sensor^35,36^, and regulates inflammatory status and cell growth^18,37^. Metformin treatment has been reported to activate AMPK activation, which inhibits fibrosis in kidney, heart, and liver^38–40^. We evaluated the efficacy of metformin and AICAR on the AMPK activity on cell growth, anti-fibrotic effect gene expression, and protein expression and cellular alignment. Our results demonstrated that AICAR and metformin inhibit cell proliferation in SSCT cells treated with PDGF-AA, and also suppressed Smad3 expression. Furthermore, we evaluated the effect of metformin in anti-fibrotic *ex vivo* experiments using 3D culture insert. Metformin treatment increased Col I production and decreased Col III in *ex vivo* culture indicating a shift to a less fibrotic state^6^. In contrast PDGF-AA treatment decreased Col I and increased Col III. The ratio of Col I to Col III is a measure of pathology of SSCT fibrosis^6^. In skin, Col I and Col III exist in a ratio of approximately 4:1, however, the ratio alter 2:1 when hypertrophic and immature scars^41^. Chikenji and colleagues reported that the ratio of Col I to Col III was 4:1 in normal SSCT tissue and nearly 1:1 in SSCT of CTS patients^6^. Furthermore, Oh *et al*. reported that, in SSCT of CTS patients, the diameter of the collagen fibrils was larger and the density of the collagen fibrils was lower than normal SSCT^42^. These results indicate that the alteration of the ratio of Col I to Col III with thickening and fragility of SSCT in CTS patients may result from increased fibrotic SSCT cells and hyperactive PDGFR signaling resulting in altered ratios of Col III and Col I, along with an overall increase in ECM. Interestingly, metformin improved the ratio of Col I to Col III above 1.0, similar to the ratios found in normal SSCT^6^. Metformin treated cells also decreased F-actin expression, which is a stress fiber, and resulted in aligning along cell shape and orientation as compared to SSCT cells with and without PDGFA treatment. Decreasing F-actin by treatment with metformin in this study may occur by activating AMPK. Previously AMPK activation has been shown to reduce F-actin expression via phosphorylation of RhoA^43^, and thereby inhibits cell spreading and migration by attenuation of lamellipodia^44^. The increasing actin stress fiber upregulates mechanosensitivity and the subjected mechanical stress differentiated into activated fibroblast/myofibroblast^45,46^. The reduction in stress fibers by activation of AMPK may attenuate mechanosensitivity in SSCT cells, suggesting that metformin may reduce CTS progression by mechanical stress, which is caused by friction force on SSCT during wrist and finger motion^47,48^.

Several non-surgical treatments are available for management of CTS. Corticosteroid therapy has been reported to have efficacy for CTS patients. However, surgical treatment is preferred because the longer lasting effect than non-surgical treatment^4^. In this study, we demonstrated that metformin has a possibility to improve the SSCT fibrosis in CTS patients and may also prevent development of CTS. Metformin has been widely used to treat type 2 diabetic patients. Diabetic patients are known to have a high risk for complication of CTS, and with a 1.51 odds ratio, however, the recipients of metformin decreases the odds ratio to 1.20^49^. Although the pathogenic mechanism might differ between diabetic and idiopathic CTS, our results provide a possibility to treat the idiopathic CTS patients by an existing FDA-approved drug.

While future work will investigate the efficacy of therapeutics *in vivo*, limitations of this study include the evaluation of chemical inhibitors *in vitro*. However, the use of the 3D system model mimics cell-cell and cell-ECM interaction closer to *in vivo* responses as compared to 2D model. Further, the 3D culture provides a suitable model as it allows for the transport and diffusion throughout, similar to what occurs *in vivo as* compared to 2D cultures^22,23^, and is often used for drug screening^50^.

**Figure 5:**
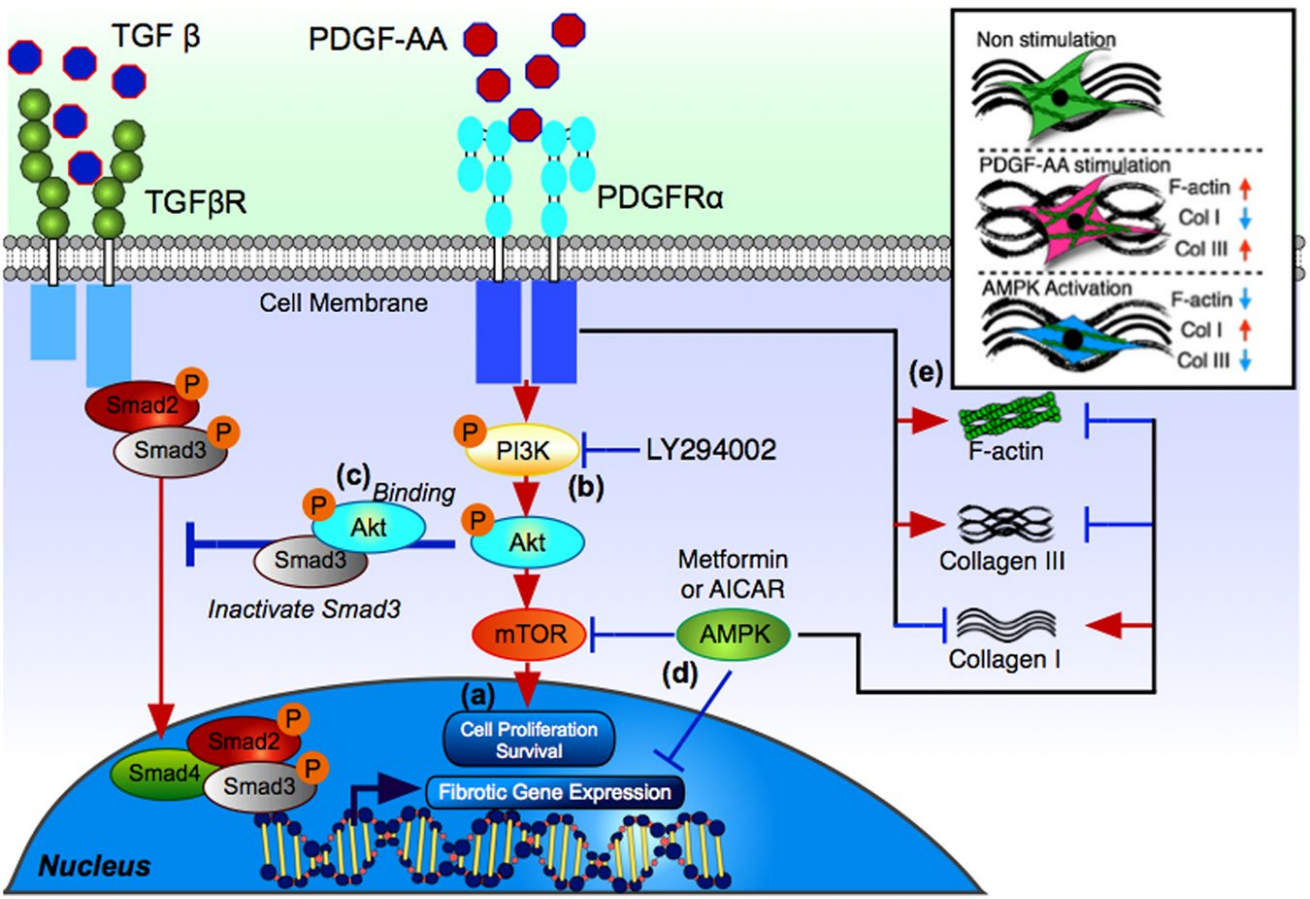
Proposed mechanism of PDGFR signaling and the effect of inhibitors in SSCT cells. PDGF-AA binds to PDGFRa receptors. LY294002 inhibits PI3K signaling (**a**) and cell proliferation (**b**). Akt phosphorylation is inhibited and modulates Smad3 activity (**c**). Metformin and AICAR activate AMPK (**d**) inhibiting both fibrotic gene/protein expression and cell proliferation. AMPK also decreased F-actin and altered the ratio of Col I to Col III (**e**).

This work has shown that PDGFRα may play an important role in mediated the pathological fibrosis found in the SSCT of CTS patients and supports the role of targeting PDGFRα in the pathogenesis of CTS. We further showed that PI3K/Akt/mTOR signaling pathway is an important therapeutic target and that AMPK activation might be an effective therapeutic approach to improve the SSCT fibrosis in CTS patients rather than targeting PI3K inhibition alone (Fig. 5). AMPK activation and the potential use of metformin, an FDA-approved drug, may provide an important prevention and/or treatment therapeutic approach for CTS therapeutics.

## Methods

### Human SSCT sample biopsy

Experiments using human samples were approved by institutional review board (IRB) at Sapporo Medical University (Approval number; 24-138), and all experiments were performed in accordance with relevant guidelines and regulations. All patients gave written informed consent. We obtained SSCT surrounding the flexor digitorum superficialis tendon from 6 idiopathic CTS patients (mean age; 70.5, range; 45-82, female). Due to ethical concerns taking tissue from healthy donors is not feasible, therefore control/normal tissue from a patient without CTS (47 years old, male) who underwent the flexor tenolysis at the carpal tunnel and 2 cadavers (86 and 94 years old, male) as control.

### Immunohistochemistry of tissue

For immunohistochemistry studies tissue was fixed in 4% paraformaldehyde overnight. The following day, the tissue was transfer to 20% sucrose in phosphate buffer and incubate overnight, and OCT embedding tissues were frozen in liquid nitrogen and store at −80 °C until use. Cryosections (8 µm thickness) were cut by cryostat. The sections were incubated in 0.01 M PBS containing 0.3% Triton-X (PBS-T) and treated with 2% bovine serum albumin (BSA) for 60 min at room temperature (RT). After washing with 0.01 M PBS-T, the sections were incubated with primary antibodies at 4 °C overnight, followed by secondary staining. Primary antibodies used were anti PDGFRα (1:200; SC-338, Santa Cruz Biotechnology, Santa Cruz, CA, USA). Secondary antibodies used Alexa Fluor 647 conjugated IgG (1:800; 111-606-003, Jackson immunoResearch, West Grove, PA, USA). Nuclei were stained using 4’,6-diamidino-2-phenylindole (DAPI) (1:1000; D523, Dojindo, Kumamoto, Japan). Sections were observed with a confocal laser scanning microscopy (Nikon/A1; Nikon, Tokyo, Japan).

### Cell preparation and proliferation assay

The SSCT from CTS patients were digested in collagenase for 60 min at 37 °C. The digested SSCT slurries were filtered through a 100 µm cell strainer (EASYstrainer^TM^ Cell; Greiner Bio-One, Kremsmuenster, Austria), and through a 40 µm cell strainer (Greiner Bio-One). Cells were suspended in the growth medium consisting of α-modified Eagle’s medium (α-MEM; Invitrogen, Grand Island, NY, USA) supplemented with 10% fetal bovine serum, 100 U/mL penicillin, and 100 μg/mL streptomycin, and incubate at 37 °C in 5% CO_2_. Cells were used in all experiments at passage 3 to 5.

For PDGF-AA stimulation and PDGFR inhibition, cells were treated with or without 10 ng/mL PDGF-AA (712402, BioLegend, San Diego, CA, USA), combination of 10ng/mL PDGF-AA and 1 µM or 10 µM Imatinib (I0906, Tokyo Chemistry Industry Co., Tokyo, Japan), or 25 µM LY294002(70920, Cayman Chemical, Ann Arbor, MI, USA), or 10 µM U0126(70970, Cayman Chemical), or 0.5 mM AICAR (10010241, Cayman Chemical), or 10 mM 1,1-Dimethylbiguanide hydrochloride (metformin) (D150959, Sigma-Aldrich, St. Louis, MO, USA) for 2days as per experimental design. These doses of inhibitor decided that based on previous study, which indicated specific inhibition each signaling and did not affect cell viability^11,40,51^. WST-8 assay was performed to assess the proliferation ability of the cells by using the Cell Counting Kit-8 (CK04, Dojindo) as previously described^52^.

### Immunocytochemistry of cultured cells and Flow Cytometry

Cells were cultured on 8-well chamber slide (Thermo Scientific^TM^ Nunc^TM^ Lab-Tek^TM^; Thermo Fisher Scientific, Waltham, MA). Cultured cells were fixed with 4% paraformaldehyde for 15 min at RT and then incubated in PBS-T and treated with 2% bovine serum albumin (BSA) for 60 min at RT. After washing with PBS-T, the cells were incubated with primary antibodies at 4 °C overnight, followed by secondary staining. Primary antibodies used were anti phosho-Akt (Ser473) (1:200; Poly6490, Biolegend), phosho-AMPK alpha-1/2 (Thr183/Thr172) (1:100: bs-4002R, Bioss Antibodies, Boston, MA), anti-TGF-β1(1:100; ab92486, Abcam), and anti Smad2/3(D7G7) (1:1600, CST Signaling Technology). Secondary antibodies used Alexa Fluor 488 conjugated IgG (1:800; 705-545-003, Jackson immunoResearch) or Alexa Fluor 647 conjugated IgG (1:800; 111-606-003, Jackson immunoResearch). Nuclei were stained by 4′,6-diamidino-2-phenylindole (DAPI) (1:1000; Dojindo). Actin filaments were stained by CytoPainter Phalloidin-iFluor 488 Reagent (1:1000, ab176753, Abcam, Cambridge, UK) according to the manufacture’s instruction. These slides were observed with a confocal laser scanning microscopy (Nikon/A1) and fluorescence microscopy (BIOREVO BZ-9000, Keyence Corp., Osaka, Japan). To quantify the TGF-β1 expression, the fluorescence intensity was measured by using Fiji(ImageJ, National Institutes of Health) and fluorescence intensity of TGF-β1 was normalized by number of cells stained by DAPI. To quantify the Smad2/3 expression, the fluorescence intensity in nuclei and cytoplasm were measured by using Fiji, and calculated the fluorescence intensity ratio of nuclei to cytoplasm.

For flow cytometry analysis, cultured cells were trypsinized and resuspended in in PBS with 2% FBS and 5 mM EDTA, and stained with primary antibody for 30 min at 4 °C. Primary antibodies used were anti PDGFRα (1:200; SC-338, Santa Cruz). Secondary antibodies used Alexa Fluor 647 conjugated IgG (1:400; 111-606-003, Jackson immunoResearch) for 30 min at 4 °C in the dark. Stained cells were analyzed by FACSCanto^TM^ II (BD Biosciences).

### In-Cell ELISA

Cells were cultured on 96-well plate, and treated with or without 10 ng/mL PDGF-AA, and combination of 10ng/mL PDGF-AA and 10 mM metformin for 2 days. Cultured cells were fixed with 4% paraformaldehyde for 15 min at RT and then incubated in PBS-T and treated with 2% bovine serum albumin (BSA) for 60 min at RT. After washing with PBS-T, the F-actin was stained by CytoPainter Phalloidin-iFluor 488 Reagent (1:1000; Abcam) according to the manufacturer’s guidelines. Nuclei were stained by 4′,6-diamidino-2-phenylindole (DAPI) (1:1000; Dojindo). The fluorescence intensity was measured by microplate reader (INFINITE M1000 PRO, Tecan Trading AG, Switzerland), and fluorescence intensity of F-actin was normalized by DAPI intensity.

### RNA extraction and Quantitative Real-Time PCR

Total RNA was isolated from cultured cell using Tri Reagent^®^ (Molecular Research Center, Inc., Cincinnati, OH), and the RNA were reverse transcribed into cDNA using a Omniscript RT Kit (205113, QIAGEN, Hilden, Germany). Quantitative PCR was performed with Power SYBR® Green Master Mix (4368702, Applied Biosystems, Foster City, CA, USA) using Applied Biosystems 7500 Fast Real-Time PCR System (Applied Biosystems) under the following cycling conditions: 50 °C for 2 min and 95 °C for 10 min followed by 40 cycles of amplification (95 °C for 15 s and 60 °C for 1 min). Expression levels were normalized to gluceraldehyde-3-phosphate dehydrogenase (GAPDH). Specific primer sequences used for PCR are listed in Table 1. ΔΔCt method was used to compare each data^53^. 2 All work was performed with 2 internal replicates and 2 experimental replicates. Gene expression results are reported normalized to control untreated SSCT cell expression.

**Table 1:** Specific primer sequence used for real-time PCR.

Gene	Forward	Reverse	Size	Ascension Number
TGF-β	5′ GTGGAAACCCACAACGAAAT 3′	5′ CGGAGCTCTGATGTGTTGAA 3′	83	NM_000660.6
CTGF	5′ TCCCACCCAATTCAAAACAT 3′	5′ TGCTCCTAAAGCCACACCTT 3′	144	NM_001901.2
COL1A2	5′ TCCAAAGGAGAGAGCGGTAA 3′	5′ CAGATCCAGCTTCCCCATTA 3′	112	NM_000089.3
COL3A1	5′ CCAGGAGCTAACGGTCTCAG 3′	5′ CAGGGTTTCCATCTCTTCCA 3′	103	NM_000090.3
SMAD3	5′ GGGCTCCCTCATGTCATCTA 3′	5′ TTGAAGGCGAACTCACACAG 3′	98	NM_005902.3
GADPH	5′ ATTGCCCTCAACGACCACTT 3′	5′ TGCTGTAGCCAAATTCGTTGTC 3′	64	NM_002046.5

### Evaluation of Collagen production on 3-dimentional culture membrane and F-actin alignment

To quantitate the collagen production from cells treated by PDGF-AA and metformin, cells cultured on 3-dimentional culture membrane, VECELL (Vessel Inc.). Cells were cultured in growth medium and treated with 10ng/mL PDGF-AA (Biolegend), and with or without 10 mM metformin for 1 week. The membranes were fixed in 4% paraformaldehyde for 15 min at RT and stained with primary antibodies at 4 °C overnight, followed by secondary staining. Primary antibodies used were anti collagen I (1:1000, ab34710, Abcam) and collagen III (1:1000; GTX111643, GeneTex, Los Angeles, CA, USA). Secondary antibodies used Alexa Fluor 647 conjugated IgG (1:800; 111-606-003, Jackson immunoResearch). Nuclei were stained by DAPI (1:1000; Dojindo). Actin filaments were stained by CytoPainter Phalloidin-iFluor 488 Reagent (1:1000; ab176753, Abcam) according to the manufacture’s instruction. The Z-stack images were acquired for 3D reconstruction and analyzed the volume of collagen I and collagen III with confocal laser scanning microscopy and NIS Elements (Nikon).

Data of F-actin alignment was calculated as dispersion of the F-actin angle. Dispersion is the extent to which the magnitude of the set of data differs, that is the degree of diversity, and the dispersion is known as the square of the standard deviation. F-actin angle was defined the line of F-actin which passed long axis of nucleus against the 3D scaffold alignment.

### Statistical Analysis

Normality was assessed using Shapiro-Wilk test. Pairwise t tests assessed difference percentage of PDGFRα positive cells between control tissue and CTS tissue. A One-Way analysis of variance (ANOVA) or Kruskal-Wallis test was conducted to assess the difference among each cell culture conditions. Pairwise comparisons were made only when the One-way ANOVA or Kruskal-Wallis test indicated statistical significance. *P*-values for multiple comparisons were adjusted by Holm methods. A repeated measure ANOVA was conducted to assess the concentration dependent manner of each inhibitor and activator agents. Statistical analyses were performed using EZR, which is a graphical user interface for R (The R Foundation for Statistical Computing, Vienna, Austria)^54^. Two-sided *P*-values less than 0.05 were considered statistically significant.

## Electronic supplementary material

Supplemental Figure 1
