# Supplementary material for: PDGFR Signaling Mediates Hyperproliferation and Fibrotic Responses of Subsynovial Connective Tissue Cells in Idiopathic Carpal Tunnel Syndrome
Source: Sci Rep. 2017 Nov 23;7:16192. doi: 10.1038/s41598-017-16443-w (PMC5700922; doi:10.1038/s41598-017-16443-w)
Supplement: Supplementary file 1 — Supplemental Figure 1 [file 41598_2017_16443_MOESM1_ESM.pdf]

PDGFR Signaling Mediates Hyperproliferation and Fibrotic Responses of Subsynovial Connective Tissue Cells in Idiopathic Carpal Tunnel Syndrome

Yuki Saito, M.S. [1] E-mail: yuki.saito@sapmed.ac.jp

Takako Chikenji, Ph.D. [1,*] E-mail: chikenji@sapmed.ac.jp

Yasuhiro Ozasa, M.D., Ph.D. [2] E-mail: yozasa@sapmed.ac.jp

Mineko Fujimiya, MD, Ph.D. [1] E-mail: fujimiya@sapmed.ac.jp

Toshihiko Yamashita, MD, PhD. [2] E-mail: tyamasit@sapmed.ac.jp

Anne Gingery, Ph.D. [3] E-mail: gingery.anne@mayo.edu

Kousuke Iba, M.D., Ph.D. [2] E-mail: iba@sapmed.ac.jp

1. Department of Anatomy, Sapporo Medical University School of Medicine, Sapporo, Japan.

2. Department of Orthopaedic Surgery, Sapporo Medical University School of Medicine, Sapporo, Japan.

3. Department of Orthopedic Surgery, Mayo Clinic, Rochester, Minnesota.

* Corresponding author

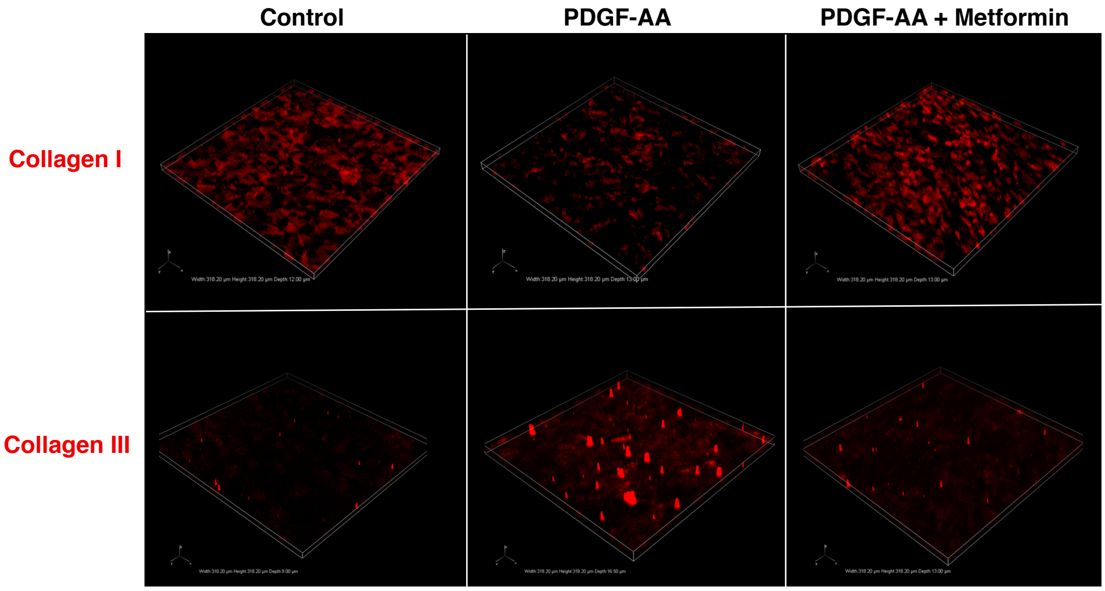

Supplemental Figure 1. Representative image of 3D reconstruction when the SSCT cells cultured on 3D culture membrane. The SSCT cells was cultured on the 3D culture membrane, VECELL (Vessel Inc.), with PDGF-AA and metformin supplementation. The SSCT cell produced the collagen I without PDGF-AA supplementation (control), while the cell treated with PDGF-AA increased the collagen III production, but decreased the collagen I production like as the condition of CTS patient in vivo (PDGF-AA). The production of the collagen III by PDGF-AA was inhibited by metformin supplementation, which increased the collagen I like as control (PDGF-AA + Metfromin).
